# The circadian clock, metabolism, and inflammation—the holy trinity of inflammatory bowel diseases

**DOI:** 10.1042/CS20256383

**Published:** 2025-07-04

**Authors:** Oren Froy, Yael Weintraub

**Affiliations:** 1 Institute of Biochemistry, Food Science and Nutrition, The Robert H. Smith Faculty of Agriculture, Food and Environment, The Hebrew University of Jerusalem, Rehovot, Israel; 2 Institute of Gastroenterology, Nutrition and Liver Diseases, Schneider Children’s Medical Center of Israel, Petach Tikva, Petach Tikva, Israel; 3 Gray Faculty of Medical and Health Sciences, Tel-Aviv University, Tel-Aviv, Israel

**Keywords:** circadian, clock, IBD, metabolism, colitis, interleukin, AMPK

## Abstract

Inflammatory bowel diseases (IBDs), including Crohn’s disease and ulcerative colitis, are characterized by relapsing-remitting immune activation and inflammation within the gastrointestinal tract. The immune system activity displays diurnal variation, which is regulated by the circadian clock. This is achieved by modulating the number of circulating lymphocytes, antibody production, cytokine production, host– pathogen interactions, and the activation of innate and adaptive immunity around the circadian cycle. Indeed, intestinal biopsies and peripheral blood cells obtained from patients with active IBD demonstrated reduced circadian clock gene expression. Key clock regulatory proteins, such as retinoic acid receptor-related orphan receptors, REV-ERBs, peroxisome proliferator-activated receptors (PPARs), PPARγ transcriptional co-activator 1α, adenosine monophosphate-activated protein kinase and Sirtuin 1, have a dual function as they regulate clock gene expression as well as the expression of certain pro- and anti-inflammatory factors through the NF-κB signaling pathway. All the aforementioned clock regulatory proteins are also key regulators of metabolism. Thus, these factors form a complex triangular network that regulates the circadian clock, inflammation, and metabolism. Emerging data support the notion that clock disruption is associated with inflammation and aberrant metabolic regulation and that regulators of the circadian clock may play a role in inflammatory and metabolic processes. In this review, we will focus on the interrelations among the circadian clock, metabolism, and inflammation in IBD.

## The circadian clock and circadian rhythms

Organisms on Earth anticipate the light–dark cycle through an internal circadian clock (circa diem, meaning ‘about a day’) that aligns with the 24 hour cycle by responding to light [1]. This clock provides a survival advantage by co-ordinating behavioral and physiological processes to follow 24 hour rhythms, ensuring they peak at optimal times of day to evade predators, locate food, and find mates. Disruption of this regulation can lead to sleep–wake disorders, neurological and psychiatric conditions, metabolic and immune imbalances, and altered hormone secretion, significantly affecting overall health [1,2]. This indicates that disruption of the internal clock, resulting in disturbed circadian rhythms, could play a role in the development of various illnesses.

**Figure 1:**
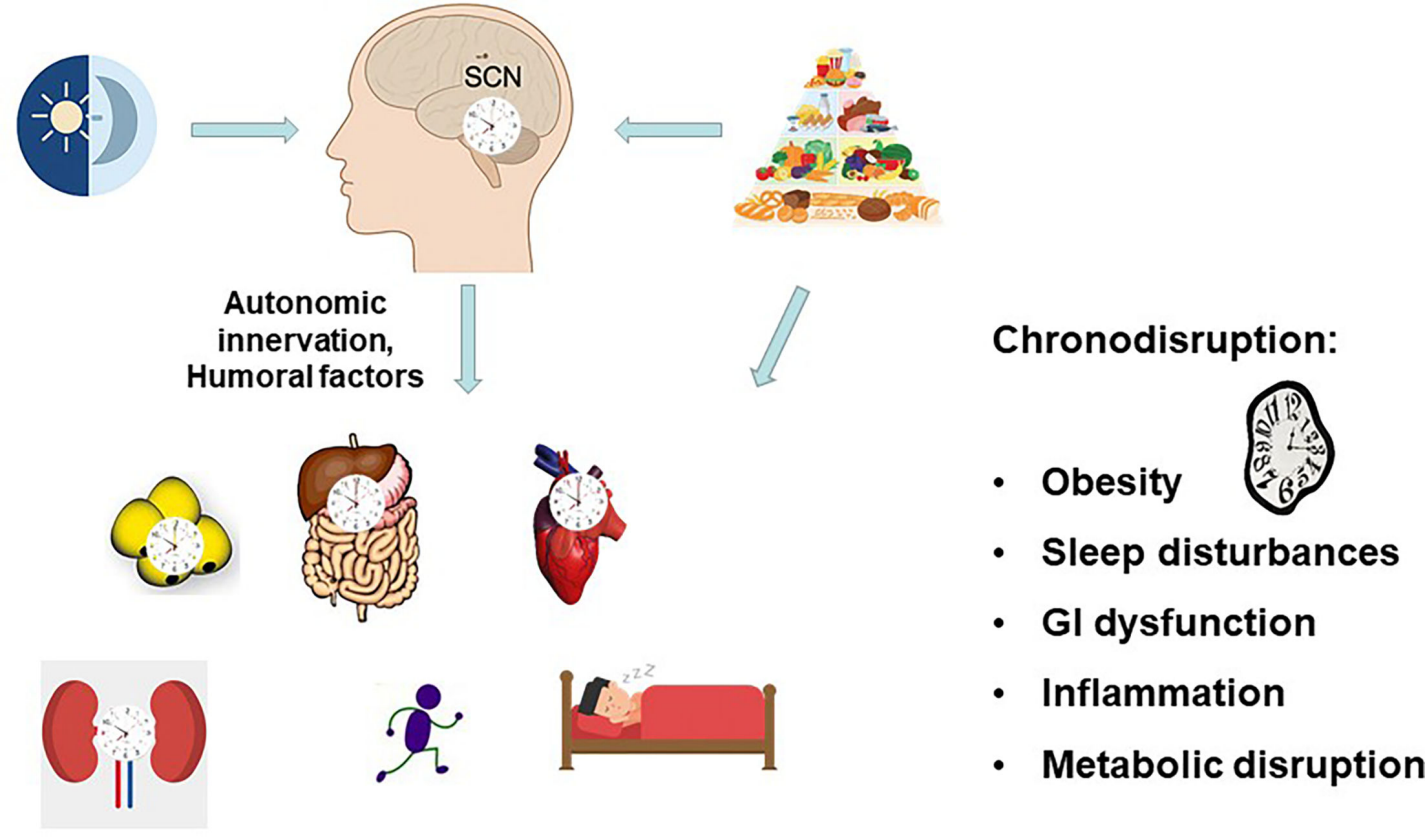
The circadian system. The SCN dictates rhythms in peripheral tissues and physiological activities, such as locomotor activity and sleep–wake cycle. Light and feeding affect either the central clock in the SCN or peripheral clocks. Chronodisruption leads to disrupted physiology.

The central circadian clock, located in the suprachiasmatic nuclei (SCN) of the hypothalamus, is regulated by environmental light (Figure 1). Light triggers a daily reset of the SCN, aligning its free-running period with the environmental light–dark cycle. Photosensitive retinal ganglion cells detect light and transmit signals via the retinohypothalamic tract to SCN neurons, which regulate central circadian outputs [1]. Peripheral clocks, resembling those in SCN neurons, are found throughout the body in various tissues, including the heart, adipose tissue, adrenal gland, pancreas, liver, intestines, and the immune system [2] (Figure 1). The central oscillator in the SCN regulates peripheral rhythms through direct neuronal pathways via the autonomic nervous system, endocrine pathways via the release of humoral factors, and indirectly by influencing rhythmic behaviors, such as feeding, locomotor activity, and body temperature, which subsequently drive rhythmic gene expression [3] (Figure 1).

**Figure 2:**
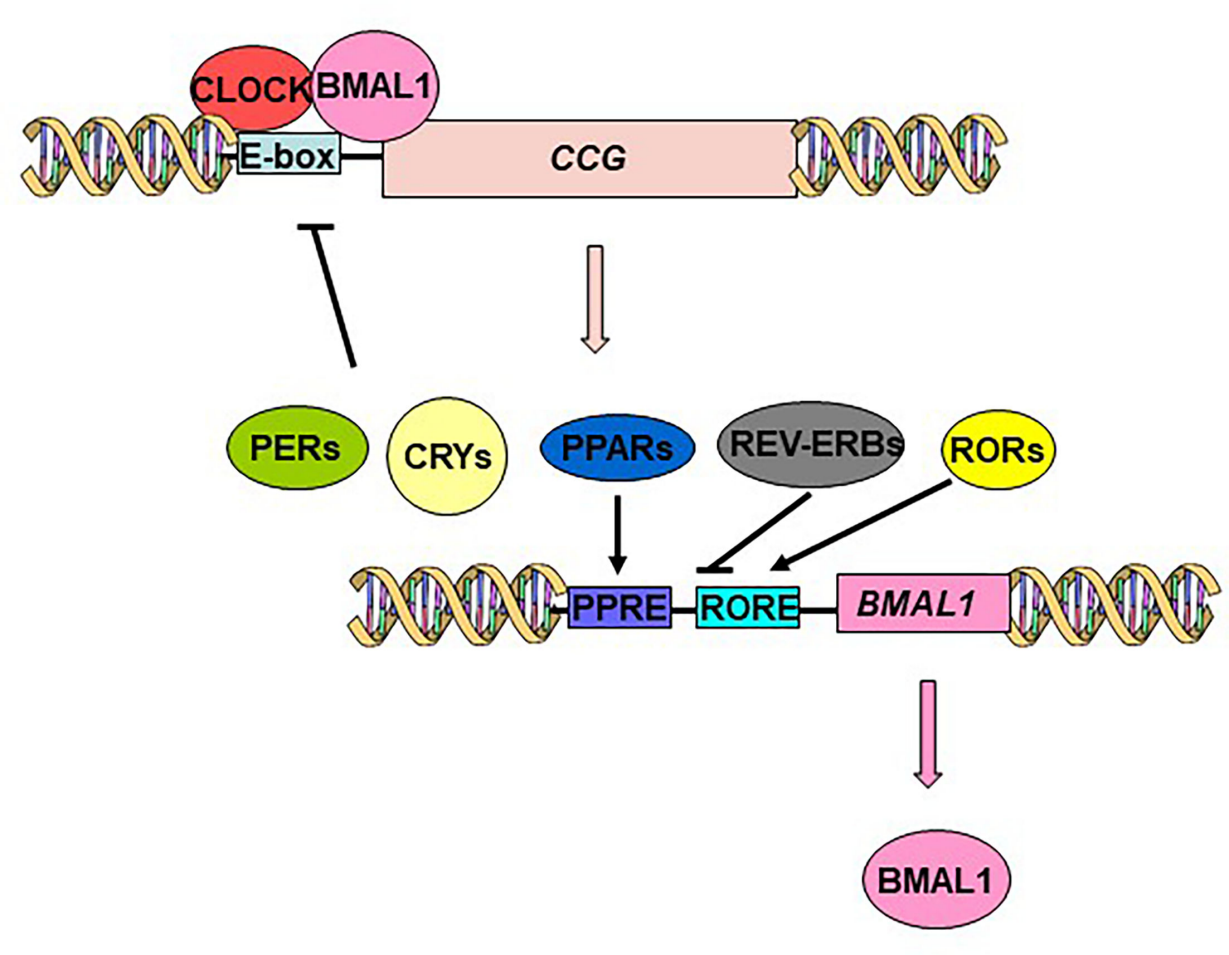
The circadian clock mechanism. The core mechanism of the mammalian circadian clock is composed of a positive limb (CLOCK and BMAL1) and a negative limb (CRYs and PERs). CLOCK and BMAL1 dimerize in the cytoplasm and translocate to the nucleus. The CLOCK:BMAL1 heterodimer binds to enhancer E-box sequences located in the promoter region of *Per* and *Cry* genes to activate their transcription. PER and CRY proteins undergo nuclear translocation and inhibit CLOCK:BMAL1, resulting in decreased transcription of their own genes. CLOCK:BMAL1 heterodimer also induces the transcription of *REV-ERBs*, *RORs* and *PPARs*. RORs, REV-ERBs, and PPARs regulate *BMAL1* expression. RORs stimulate but REV-ERBs inhibit *BMAL1* transcription, acting through ROR elements (RORE). CCG, clock-controlled genes.

Approximately 5–20% of tissue-specific genes exhibit oscillatory expression patterns, highlighting the influence of circadian regulation on diverse biological processes [2,3]. Circadian rhythms in both SCN neurons and peripheral cells are driven by intracellular transcriptional–translational feedback loops. These loops involve cis-regulatory elements, including E-boxes, D-boxes, and retinoic acid receptor-related orphan receptor elements (ROREs) [1] (Figure 2). Circadian Locomotor Output Cycles Kaput (CLOCK) and brain and muscle ARNT-like protein 1 (BMAL1) serve as the primary transcription factors of the molecular clock. Together, CLOCK and BMAL1 form a heterodimer, known as the CLOCK:BMAL1 complex, which activates transcription by binding to E-box (5′-CACGTG-3′) and E-box-like promoter sequences [3]. The PERIOD (*PER1*, *PER2*, *PER3*) and CRYPTOCHROME (*CRY1* and *CRY2*) genes are among the regulatory targets of CLOCK:BMAL1 (Figure 2). Translation, oligomerization, nuclear translocation, and the binding of PERs:CRYs to the CLOCK:BMAL1 heterodimer result in the inhibition of transcription, thereby establishing the negative feedback loop (Figure 2). In the second key transcriptional loop, the CLOCK:BMAL1 heterodimer promotes the transcription of the nuclear receptor genes REV-ERBα (also called NR1D1) and REV-ERBβ. These nuclear receptors suppress BMAL1 expression, while RORα and RORγ enhance its expression through the RORE (Figure 2). All the previously mentioned genes show 24 hour oscillations in peripheral tissues [3].

## Circadian control of gastrointestinal tract function

A wide variety of processes in the GI tract and its accessory digestive organs display 24 hour rhythmicity. The circadian clock has been proposed as the mechanism that controls digestive and metabolic processes by co-ordinating simultaneous activity of different tissues [4]. Carbohydrate digestion and metabolism require the co-ordination of clocks in the brain, liver, pancreas, and intestine. Similar multiple-organ co-ordination networks exist in lipid digestion and bile acid metabolism and the digestion of peptides and absorption of amino acids [4]. In addition, circadian rhythms exist in digestive enzyme production and secretion and nutrient absorption in the small intestine. The colon also demonstrates circadian timing in electrolyte absorption, barrier function, motility, and even fecal defecation [4]. Circadian rhythms are also seen in gut tissue self-renewal, including migration of cells upward from the crypts of Lieberkühn and proliferation of mucosal cells [5]. Indeed, *Bmal1* regulates stem cell signaling pathways, reiterating the link between the circadian system and stem cell activity [6]. A bidirectional interaction exists between the central clock and peripheral clocks in the digestive system. On the one hand, SCN-driven mechanisms have temporal control of feeding, mostly overlapping with the active phase throughout the day. On the other hand, food intake is a significant entraining cue for the clocks in the digestive tract [7] (Figure 1). In animals and humans, unusual feeding time produces a disruption of the circadian system, which leads to unhealthy consequences, such as obesity and impaired glucose tolerance [8]. It is believed that temporal feeding restriction (also known as time-restricted feeding or eating [TRF or TRE, respectively]) can change the phase of circadian gene expression in peripheral cells by up to 12 h while leaving the phase of cyclic gene expression in the SCN unaffected [7]. The timing of feeding can synchronize the digestive tract clock independently of the central oscillator in the SCN, i.e., food intake is a more potent synchronizer than photic input in these tissues [7]. This is specifically true for the liver, which carries out many important metabolic processes.

It has been shown that the intestinal microbiota undergoes diurnal compositional and functional oscillations. These oscillations are controlled by the timing of food intake and the composition of the diet [9]. This, in turn, creates rhythms that affect locally the intestines and distally the liver [9,10]. In addition, it was shown that diurnal regulation of gut immunity depends upon the expression of the major histocompatibility complex II (MHCII), induced by a population of commensal bacteria adherent to the small intestine. MHCII expression was found to be diet dependent [9]. Thus, the immune system reacts to these daily changes in microbiome levels, thereby contributing to the complexity of the circadian host–microbiome axis.

## GI function and chronodisruption

Chronodisruption refers to the ongoing misalignment between the body’s internal rhythms and external environmental signals. This lack of synchronization can result in negative health effects due to the disturbance of internal physiological functions [11]. Chronodisruption can lead to a wide variety of alterations in GI function (Figure 1). Chronodisruption impairs gastric emptying and intestinal motility, leading to symptoms such as bloating, constipation, or diarrhea [4]. It can also interfere with the timing and efficacy of digestive enzyme secretion, resulting in suboptimal digestion [4]. Indeed, *Per1*/*Per2* double knockout mice showed no daily rhythm of fecal pellet output, total colonic pressure, and cholinergic agonist sensitivity, suggesting the role of the *Period* genes in daily colonic motility [12]. In addition, it was found that *Wee1*, the cell cycle regulator, is clock-regulated and is disrupted in *Per1/2*-deficient mice [13], supporting the role of the circadian clock in the proliferation of mucosal cells. Moreover, nutrient absorption becomes dysregulated, which may contribute to metabolic disorders such as obesity and insulin resistance [8]. The gut microbiota loses its rhythmicity under chronodisrupted conditions, fostering a pro-inflammatory state and promoting dysbiosis that promotes glucose intolerance, obesity, and other manifestations of the metabolic syndrome [9,10]. Furthermore, chronic circadian misalignment has been linked to an increased risk of gastrointestinal (GI) cancers, likely due to disrupted cell cycle control and impaired DNA repair mechanisms [14]. In summary, the integrity of GI function is closely tied to circadian homeostasis, and disturbances in this system can have widespread metabolic, inflammatory, and neoplastic consequences.

## Circadian control of metabolism

**Figure 3:**
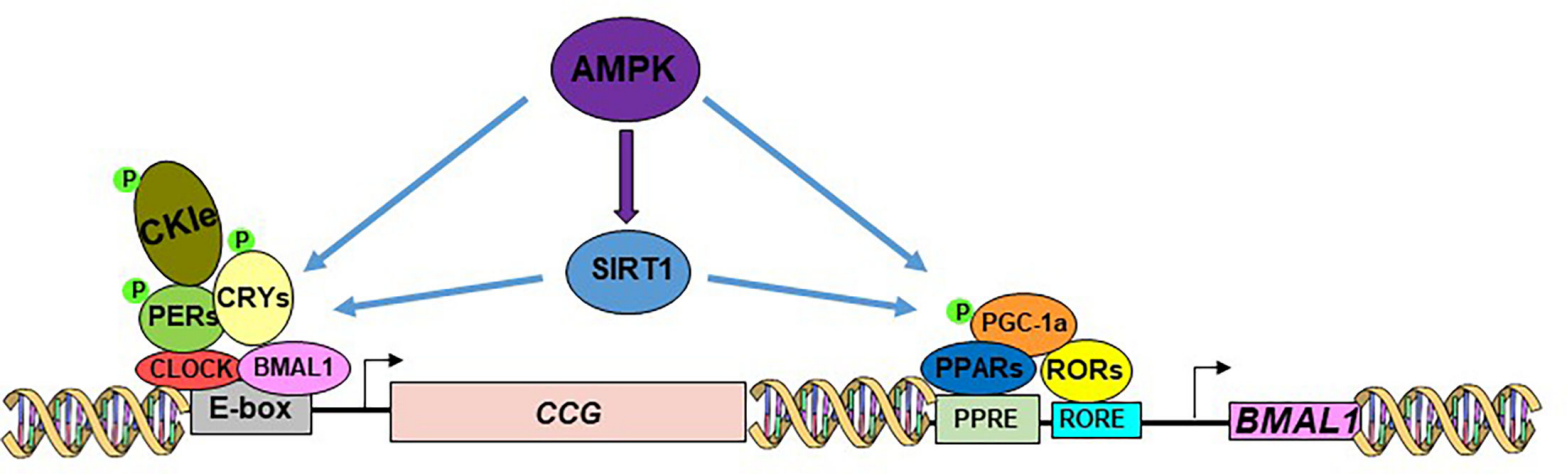
The relationship between the core clock mechanism and metabolic factors. AMPK activates SIRT1 to deacetylate the PERs and BMAL1, relieving PERs:CRYs repression. RORs need a co-activator, PGC-1α, which is phosphorylated by AMPK. SIRT1 activation leads to PGC-1α deacetylation and activation. CCG, clock-controlled genes.

The robust expression of clock genes in peripheral tissues enables the synchronized activity of nuclear receptors, enzymes, hormones, and transporters that regulate carbohydrate, lipid, and protein metabolism [3]. Metabolism also plays a role in regulating the circadian clock, achieved through the involvement of essential catabolic and anabolic factors in the core clock mechanism (Figure 3).

### AMP-activated protein kinase

AMP-activated protein kinase (AMPK) serves as a cellular energy sensor, and its activation stimulates catabolic processes. It phosphorylates and activates casein kinase I ε (CKIε), which, in turn, phosphorylates the PER proteins, marking them for degradation [15]. AMPK phosphorylates CRY1, leading to its destabilization [16] (Figure 3). Metformin, a widely prescribed medication for type 2 diabetes, indirectly activates AMPK, which results in changes to circadian rhythms [17].

### Sirtuin 1

Sirtuin 1 (SIRT1), a widespread histone deacetylase, functions as a catabolic factor in the process of transcriptional silencing [18]. SIRT1 has been linked to a range of processes, such as metabolism, stress response, protein synthesis, genomic instability, neurodegeneration, DNA damage repair, and inflammation [19]. SIRT1, activated by AMPK, deacetylates BMAL1 and PER2, resulting in PER2 phosphorylation by CKIε and its subsequent degradation, which removes the inhibition of the CLOCK:BMAL1 heterodimer [20] (Figure 3).

### Peroxisome proliferator-activated receptors

Peroxisome proliferator-activated receptors (PPARs) are nuclear receptors that interact with endogenous free fatty acids and are crucial in regulating the expression of genes that control lipid and glucose metabolism [21]. The expression of PPARs is controlled by the CLOCK:BMAL heterodimer, and in return, PPARα stimulates the expression of Bmal1 (Figure 3) by binding to the peroxisome-proliferator response element (PPRE). Indeed, PPARα agonists and *Pparγ* gene deletion have been demonstrated to affect cellular circadian rhythms in mice [22].

### REV-ERBs and RORs

REV-ERBs and RORs are two key families that connect the core clock mechanism to lipid metabolism. As noted earlier, the CLOCK:BMAL1 heterodimer controls their expression, with REV-ERBs acting to repress and RORs to promote *BMAL1* expression [23] (Figure 2). These proteins also play a crucial role in adipocyte differentiation, lipogenesis, and lipid storage [3]. Mice lacking *Rev-erbα* exhibit increased adiposity on both a regular chow and high-fat diet, likely due to enhanced fat uptake by adipose tissue [24].

### PPARγ coactivator 1

The PPARγ coactivator 1 (PGC1) family consists of transcriptional coactivators that promote mitochondrial oxidative metabolism and exhibit circadian oscillation [25]. Additionally, PGC1α promotes the expression of *BMAL1*, *CLOCK*, *PER2*, and *REV-ERBα* by co-activating the ROR family [26] (Figure 3).

In summary, clock components such as BMAL1, PERs, CRYs, RORs, and REV-ERBs regulate the rhythmic expression of genes involved in energy metabolism. PPARs, activated through PPREs, interact with PGC-1α, which enhances mitochondrial biogenesis and energy expenditure. AMPK and SIRT1 modulate circadian gene expression, and CKIε influences PER proteins’ stability and nuclear localization. Together, these factors form a tightly regulated feedback loop that aligns cellular metabolism with circadian time, thereby co-ordinating energy homeostasis with the day-night cycle [3]. Key results are summarized in Supplementary Table S1.

## Metabolic effects of chronodisruption

The relationship between the key metabolic factors discussed earlier and the underlying clock mechanism may help explain the detrimental effects of metabolic chronodisruption (Figure 1).

Mice with a mutated *Clock* gene display an altered feeding pattern, become obese, and develop a metabolic syndrome marked by increased glucose, lipid, and leptin levels, along with fat accumulation in the liver [1]. In a similar manner, *Per2* knockout mice develop significant obesity when given a high-fat diet [27]. Mice with a *Bmal1* knockout exhibit reduced diurnal variations in triglyceride and glucose levels, along with impaired gluconeogenesis and disrupted circadian rhythms in insulin activity [28]. Conditional loss of *Bmal1* function in mouse liver leads to increased oxidative stress and elevated plasma triglyceride and cholesterol levels [29]. The deletion of *Bmal1* specifically in adipocytes led to obesity in mice [30]. In mice, the knockout of *Cry1/2* similarly resulted in changes to gluconeogenesis, heightened susceptibility to obesity induced by a high-fat diet, increased insulin secretion, enhanced lipid storage in adipose tissue, and elevated levels of pro-inflammatory cytokines [31].

The modern lifestyle subjects individuals to daily chronodisruption due to artificial light exposure, shift work, and jetlag. In a laboratory experiment simulating circadian misalignment, adult participants experienced a consistent reduction in leptin levels, elevated glucose despite higher insulin, disrupted daily cortisol rhythms, and increased mean arterial pressure. Some participants also showed postprandial glucose responses that were within the typical range for prediabetes [32]. Shift workers were found to have impaired beta cell function and insulin sensitivity, resulting in impaired glucose tolerance. Furthermore, night shift work is associated with an increased risk of diabetes and cardiovascular disease [33]. Finally, in a population-based longitudinal study, individuals with higher social jetlag scores were found to have a greater average BMI, more fat mass, and a higher likelihood of being obese or meeting the diagnostic criteria for metabolic syndrome [34]. Key results are summarized in Supplementary Table S1.

## Circadian control of the inflammatory response

Immune processes, such as migration of white blood cells (WBC), the inflammatory response, host–pathogen interactions, and activation of the innate and adaptive immunity display daily fluctuations and have been shown to be regulated by the circadian clock [35]. The migration of WBCs from the bloodstream into tissues relies on the cyclic expression of adhesion molecules, such as intercellular adhesion molecule 1 (ICAM1), vascular cell adhesion molecule 1 (VCAM1), and selectins present on both endothelial cells and the surface of WBCs [36]. In addition, the circadian clock regulates the inflammatory response of macrophages, which are a primary origin of pro-inflammatory cytokines [37]. Mouse peritoneal macrophages exhibit increased expression of inflammatory cytokines, such as interleukin 6 (IL-6), and chemokines when isolated during the end rather than the beginning of the inactivity phase. As a result of the circadian changes in the immune functions, the susceptibility of the immune system to bacterial, viral, and parasitic infections fluctuates around the circadian cycle [38]. This daily variation in susceptibility correlates with the pro-inflammatory nuclear factor kappa-light-chain-enhancer of activated B cells (NF-κB) activation [38].

**Figure 4:**
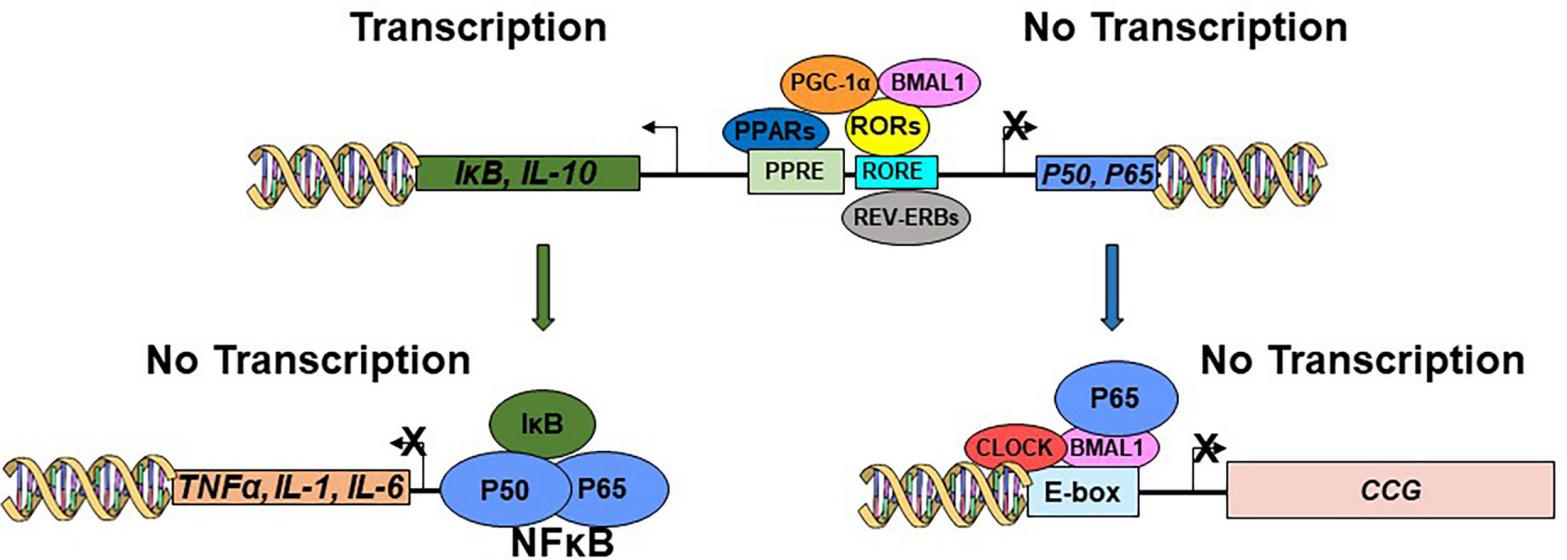
The relationship between the core clock mechanism and inflammatory processes. In the nucleus, the clock machinery induces the expression of P65, P50, IκB and IL-10. P65 inhibits the CLOCK:BMAL1 heterodimer. IκB inhibits the NF-κB from inducing the expression of the pro-inflammatory factors TNF-α, IL-1, IL-6. CCG, clock-controlled genes.

NF-κB is a transcription factor that regulates the expression of genes involved in inflammation, immunity, cell proliferation, and apoptosis [39] (Figure 4). In the canonical pathway, NF-κB, made of the p50 and p65 subunits, is normally sequestered in the cytoplasm by the inhibitor of κB (IκB) proteins. Upon activation by various stimuli, such as pro-inflammatory cytokines (e.g., tumor necrosis factor α (TNF-α), interleukin 1β (IL-1β)), microbial products (e.g., lipopolysaccharide [LPS]), or cellular stress, IκB is phosphorylated and targeted for degradation, allowing NF-κB to translocate to the nucleus and activate transcription [39] (Figure 4). In addition to its immune regulatory functions, NF-κB regulates clock functions [40]. It was demonstrated, using genetic and pharmacological methods, that disruption of the NF-κB subunit p65 resulted in altered clock gene expression. When p65 was activated, it led to a reduction in the 24 h period length and amplitude of clock gene expression, whereas its inhibition resulted in an increase in period length and induced changes in amplitude of gene expression. At the molecular level, p65 repressed the transcriptional activity of CLOCK:BMAL1 by binding to the transactivation domain of BMAL1 [40] (Figure 4). In addition, CLOCK was found within protein complexes together with the p65 subunit of NF-κB [41]. These findings establish a molecular connection between the circadian clock and immune functions (Figure 4). Key results are summarized in Supplementary Table S1.

## Inflammatory effects of chronodisruption

Circadian misalignment can directly affect the daily rhythms of immune parameters, potentially impairing the host capacity to effectively respond to pathogens or tissue damage. Additional deleterious effects may arise from an imbalance between anti-inflammatory and pro-inflammatory processes, which can result in either immune suppression or promotion of a pro-inflammatory state favorable to the development of chronic inflammatory conditions (Figure 1) [42].

Several animal models with targeted impairment in core clock genes demonstrate this association. Mouse *Bmal1* deletion led to the disruption of rhythmic fluctuations in both leukocyte and immature hematopoietic cell counts in peripheral blood [43]. Similarly, targeted removal of mouse *Bmal1* within myeloid cells resulted in the elimination of daily fluctuations in circulating inflammatory Ly6C^high^ monocytes [44]. In mouse neutrophils, targeted removal of BMAL1 resulted in the elimination of daily fluctuations in granule content and impairment in the formation of neutrophil extracellular traps that bind pathogenic microbes [45]. Similarly, NF-κB activation and induction of pro-inflammatory cytokines upon LPS challenge were reduced in *Clock*-deficient mice compared with wildtype controls [41].

The nuclear receptors REV-ERBα and RORα, which regulate BMAL1 expression, have been shown to exert mostly anti-inflammatory effects [46]. Macrophages extracted from mice lacking the REV-ERBα gene exhibited high pro-inflammatory reactions and a lack of circadian rhythm in the IL-6 response, indicating the absence of circadian regulation [46]. Similarly to *Rev-erbα* deficient mice, mice lacking *Rorα* displayed heightened vulnerability to LPS-induced lung inflammation, elevated neutrophil counts, and high levels of pro-inflammatory cytokines, such as IL-1β and IL-6, in the bronchoalveolar lavage, in comparison with their wildtype counterparts [47]. This is emphasized as the anti-inflammatory effect of RORα is manifested by upregulating IκBα, the inhibitor of NF-κB, through binding to RORE elements and by decreasing p65 nuclear translocation [48].

Evidence regarding the inflammatory effects of chronodisruption is also manifested in humans. Shift workers had significantly elevated markers of systemic inflammation, including C-reactive protein (CRP) and the cytokines TNF-α, IL-6, and IL-1β, as well as an increased number of total leukocytes, neutrophils, monocytes, and lymphocytes than daytime workers [49]. Furthermore, as food intake is a significant entraining cue for the circadian rhythm, Ramadan fasting, a form of TRE, practiced by millions of Muslims annually worldwide, has been shown to suppress pro-inflammatory cytokine expression (e.g., TNF-α, IL-6, IL-1β) and decrease circulating levels of leukocytes [50]. Other studies show that Ramadan was associated with worsening of clinical parameters. The effect was more pronounced in older patients and those with higher baseline calprotectin levels. However, Ramadan was not associated with an adverse effect on objective inflammatory markers (CRP and calprotectin) [51]. Additional studies demonstrated a decrease in TNF-α and leptin levels in adults practicing different forms of TRE [52]. Key results are summarized in Supplementary Table S1.

## Inflammatory bowel diseases

Inflammatory bowel diseases (IBDs) comprise two types of chronic idiopathic intestinal inflammatory disorders: Crohn’s disease (CD) and ulcerative colitis (UC) [53,54]. The pathogenesis is multifactorial, involving genetic predisposition, epithelial barrier defects, dysregulated immune responses, dysbiosis, and environmental factors. In CD, all segments of the GI tract can be affected, the most common being the terminal ileum and colon. Inflammation is typically segmental, asymmetrical, and transmural. Patients with UC, on the other hand, develop mucosal inflammation starting in the rectum that can extend continuously to proximal segments of the colon [53,54].

Population-based studies demonstrate the prevalence of IBD in North America and many European countries to exceed 0.3%, whereas incidence in these areas is stabilizing. Conversely, in newly industrialized countries whose societies are becoming more westernized (e.g., Africa, Asia, and South America) the incidence of both CD and UC is constantly rising [55]. The incidence and prevalence of pediatric IBD are continuing to climb as well, with data emerging from regions where rates were previously not reported, emphasizing IBD is a global 21st-century disease [56]. Various environmental exposures have been found to contribute to the development of IBD, including: smoking, antibiotic use, childbirth mode, breastfeeding, dietary habits, sedentary behavior, air pollution, vitamin D deficiency, urban environments, and microbial dysbiosis [57]. Furthermore, changes in sleeping and eating patterns and circadian disruptions have been linked to IBD [58].

IBD is characterized by alternating periods of clinical remission and recurrence. However, there is a dissociation between clinical symptoms and mucosal disease activity. Persistent subclinical inflammation occurring during clinical remission is thought to lead to complications, progressive bowel damage, and increased risk of malignancy [53,54]. Most current treatment strategies do not appear to significantly alter the natural course of the disease, although reduction in the need for surgery or the occurrence of neoplasia has been reported over time [59].

The association between IBD and metabolic disorders has been pointed out in clinical research lately. A recent meta-analysis found a positive association between metabolic syndrome and UC compared with CD. In addition, patients with IBD are at increased risk for acute arterial events, likely due to systemic inflammation [60]. Furthermore, there is considerable evidence suggesting that obesity adversely affects the response to IBD treatment, especially with biologics, such as TNF-α antagonists, and immunomodulators, such as azathioprine and 6-mercaptopurine [60]. The mainstay of current IBD therapy has been shown to positively affect metabolic impairments as well, as anti-TNF-α treatment decreases LDL and increases HDL cholesterol levels, reduces insulin resistance, improves endothelial dysfunction, and reduces cardiovascular risk [61]. All the metabolic factors mentioned above add a substantial level of complexity to the understanding of IBD pathogenesis and pose a challenge in how to translate the data into therapeutic avenues.

## Pivotal inflammatory signaling pathways in IBD

### The NF-κB signaling pathway

Dysregulated NF-κB signaling has a pivotal role in IBD as it contributes to the chronic inflammation observed in UC and CD. Increased activation of NF-κB has been observed in colonic mucosa from IBD patients, leading to the up-regulation of pro-inflammatory cytokines (e.g., TNF-α, IL-1β, IL-6), adhesion molecules and inflammatory mediators [62]. TNF-α is a hallmark of IBD, as it can further activate NF-κB, creating a vicious cycle of inflammation [63]. NF-κB activation promotes the recruitment and activation of immune cells (e.g., macrophages, neutrophils, T cells) to the intestinal mucosa, perpetuating inflammation and tissue damage. In addition, it has been shown that NF-κB signaling is an active factor in the maintenance of epithelial integrity and immune homeostasis in the gut [64].

Inhibition of NF-κB signaling represents a promising therapeutic strategy for the treatment of IBD [65]. Long-standing anti-inflammatory agents accepted as the mainstay therapeutic regimens for IBD, such as corticosteroids (e.g., prednisone, prednisolone), aminosalicylates (e.g., 5-aminosalicylic acid, mesalamine), and immunomodulators (e.g., azathioprine, 6-mercaptopurine) exert their therapeutic effects at least in part by suppressing NF-κB activation and its downstream inflammatory responses [64]. Biological therapies targeting specific components of the NF-κB pathway, such as anti-TNF-α agents (e.g., infliximab, adalimumab), anti-integrin agents (e.g., vedolizumab), and anti-interleukin agents (e.g., ustekinumab), are currently the mainstay of therapy for moderate to severe IBD [64]. Interestingly, polymorphisms in genes involved in the regulation of the NF-κB pathway (TLR2, TLR4, and NFKBIA) have been associated with response to anti-TNF therapy in IBD [66]. These current biological therapies targeting the NF-κB pathway have revolutionized the management of IBD.

### The interleukin-10 (IL-10) signaling pathway

IL-10 is an anti-inflammatory cytokine produced by various immune cells, including T cells, B cells, macrophages, and dendritic cells. IL-10 exerts its anti-inflammatory effects by binding to its receptor (IL-10R) expressed on target cells, leading to the activation of downstream signaling pathways that suppress immune responses and inflammation [67]. The IL-10 anti-inflammatory response plays a crucial role in regulating immune homeostasis and preventing excessive inflammation in the intestine. Dysregulation of the IL-10 pathway has been implicated in the pathogenesis of IBD [68]. Genetic mutations in IL-10 or IL-10R genes are associated with very early onset of IBD, highlighting the importance of IL-10 signaling in maintaining intestinal immune homeostasis [69]. It has recently been found that macrophages and tissue samples of UC patients had lower amounts of IL-10 mRNA and higher expression levels of the associated pro-inflammatory cytokines including TNF-α, IL-1β, and IL-6 compared with healthy controls, suggesting that the immune suppressive functions of the macrophages in UC patients are impaired [70].

## The AMPK-SIRT1-PGC1α axis—linking metabolism and inflammation in IBD

As mentioned above, the AMPK-SIRT1-PGC1α axis includes key metabolic factors that play a pivotal role in the clock mechanism as well (Figure 3). Dysregulation of the AMPK-SIRT1-PGC1α axis has been implicated in the pathogenesis of IBD, specifically UC [71,72]. Decreased AMPK activity and SIRT1 expression have been observed in colonic mucosa from IBD patients, leading to impaired mitochondrial function, increased oxidative stress, and inflammation [72]. Restoration of AMPK and SIRT1 activity or overexpression of PGC1α has been shown to ameliorate experimental colitis in animal models by suppressing inflammation, enhancing barrier function, and reducing oxidative damage [73].

Targeting the AMPK-SIRT1-PGC1α axis may represent a potential therapeutic strategy in IBD. Indeed, metformin, a pharmacological activator of AMPK, has demonstrated anti-inflammatory and antioxidant effects [74]. Additionally, it enhances gut barrier integrity in both cellular and animal models of IBD. Finally, metformin can restore gut microbiota in mice with colitis, eventually leading to reduced intestinal inflammation. This body of evidence suggests that metformin, a commonly used worldwide treatment for type 2 diabetes, could be a viable alternative treatment for IBD [74]. Similarly, resveratrol, a pharmacological activator of SIRT1, which has anti-inflammatory properties, has been shown to combat inflammation in chemically induced colitis [75]. It achieves this by targeting various molecular pathways, such as NF-κB, SIRT1, TNF-α, and autophagy. These promising effects observed in preclinical studies suggest that resveratrol may offer future therapeutic benefits for patients with IBD [75]. Another important avenue is the use of glucagon-like peptide 1 (GLP-1) agonists, as an association between lower risk of IBD-related surgery and GLP-1 receptor agonists was found [76]. Activation of the GLP-1 receptor activates the AMPK and inhibits the NF-κB signaling pathway [77,78].

## The interrelations among the circadian clock, metabolism, and inflammation in IBD

Epidemiological studies indicate that the westernization of diet conveys a risk of developing IBD [61]. Furthermore, macronutrients in the western diet, mainly simple carbohydrates, long-chain fatty acids, and food additives, are risk factors for developing metabolic diseases. Metabolic inflammation is triggered by the constant overexposure to these macronutrients, which activate the innate and adaptive immune response in metabolically active tissues, leading to systemic low-grade inflammation. Diet-induced stress is translated into an inflammation response *via* nutrient sensing through Toll-like receptors (TLRs), NOD-like receptors (NLRs) and ER stress sensors, leading to the activation of NF- κB signaling and/or mitogen-activated protein kinases (MAPKs), which induce the production of inflammatory cytokines, such as TNF-α, IL-1β, IL-6 and IL-8. Besides the direct immunomodulatory effects of macronutrients, experimental evidence links diet-induced gut microbiota perturbation with metabolic diseases. Depletion of butyrate-producing bacteria, consumption of saccharin (an artificial sweetener), fructose, and high-fat diets have all been related to changes in gut microbial composition and function. Microbial dysbiosis, which fuels gut inflammation and metabolic dysregulation, underlies the complex pathophysiology of metabolic inflammation [61].

Epidemiological research points out an association between disrupted circadian rhythms and IBD [79]. Individuals who work night shifts have a higher likelihood of developing IBD. Furthermore, disrupted circadian rhythms and sleep disturbances exacerbate IBD symptoms and contribute to the severity of the disease [80]. These findings imply that maintaining aligned circadian rhythms may confer a protective role against active inflammation in IBD. Indeed, clock gene disruption was identified in colitis-induced mouse models and in patients with IBD. Mice with DSS-induced colitis exhibited notable reductions in *Cry1*, *Per2*, *Npas2*, and *Rev-erbα* expression, alongside significantly increased expression of *Rorα* [46]. Patients with IBD demonstrated reduced expression of the clock genes *BMAL1*, *CLOCK*, *PER1/2*, and *CRY1/2* in both mucosal tissue and peripheral WBC [42,81]. When comparing CD and UC, it was found that UC patients exhibited notably reduced expression levels of *PER1/2* and *BMAL1* in inflamed mucosa compared with CD patients [42]. In addition, these patients exhibited significantly elevated levels of inflammation markers as well, namely C-reactive protein and calprotectin, when compared with controls [81].

The importance of intact circadian rhythmicity in the digestive tract has been reiterated in studies involving clock-specific knockout and mutant mouse models. Genetic deficiency of various circadian clock components has been shown to increase susceptibility to IBD in mice [13,46,82,83]. The loss of *Per1/2* exacerbates colitis by decreasing the population of mucus-producing secretory cells and hindering regenerative proliferation during inflammation [13]. In mice with an intestinal epithelial-specific *Rorα* knockout, colitis is exacerbated, as *Rorα* suppresses the NF-κB-mediated pro-inflammatory activity [83]. *Rev-erbα* mutant mice exhibit exacerbated colitis due to decreased activity of the *Nlrp3* inflammasome, a multi-protein complex involved in the innate immune responses and inflammation [46].

In the intestine, Wnt signaling and the Hippo pathway are crucial for the maintenance of intestinal stem cells (ISCs) and the regulation of epithelial cell renewal and differentiation promoting regeneration and mucosal healing [84]. Dysregulation of these pathways has been implicated in various diseases, including IBD [84]. Disruption of the circadian clock can interfere with epithelial regeneration. Indeed, it was found that BMAL1 exacerbates colitis severity by hindering healing and affecting the activity of the Wnt and Hippo pathways during regeneration [82].

Impairment in intestinal barrier integrity is an additional aspect in the development of IBD [85]. Tight junctions seal the spaces between epithelial cells to block the passage of luminal substances. The tight junction proteins occludin and claudin-1 exhibit circadian oscillations, which are inversely related to *Per2* mRNA expression levels [86]. Mice deficient in PER2 showed consistently increased levels of occludin and claudin-1, whereas mice lacking CLOCK exhibited consistently reduced levels of these two tight junction proteins and were more vulnerable to intestinal injury induced by DSS [86]. Therefore, it has been proposed that the circadian clock influences colonic permeability, with CLOCK and PER2 exhibiting contrasting roles in regulating the integrity of the intestinal barrier [86]. Conversely, there were no differences observed in the transcriptional expression of occludin, claudin-1, tight junction protein 1, and mucin 2, among colitis-induced mice lacking *Bmal1* and their wild type controls [82].

As mentioned above, the NF-κβ signaling pathway activates multiple processes, resulting in elevated transcription of pro-inflammatory mediators, such as IL-6, TNF-α, and interferon (IFN)-γ [62]. RORα, which regulates metabolism and the expression of core clock genes, additionally controls the expression of pro-inflammatory cytokines, including IL-6 and IL-8, through the NF-κB signaling pathway [87] (Figure 4). RORα exerts its pro-inflammatory effects by inducing the expression of the NF-κB suppressor IκBα [48]. PPARs, RORγ, and PGC1α, expression regulators of BMAL1 as well as metabolism, exert anti-inflammatory properties by suppressing pro-inflammatory gene expression, such as IL-17, IL-1β, and TNF-α (Figure 4). Indeed, PPARγ, a negative regulator of NF-κB-dependent inflammation, was found to be reduced in colonocytes of patients with UC [88]. In addition, hepatic-specific overexpression of PGC1α was found to positively enhance the IL-10-mediated anti-inflammatory response [89]. Furthermore, in a rat model of colitis and in a colonocyte cell line derived from a patient with colorectal adenocarcinoma, the activation of the metabolic/clock AMPK-SIRT1-PGC1α pathway has been shown to down-regulate the expression of pro-inflammatory cytokines [90]. Simultaneous expression of clock, inflammation, and their mutual regulatory genes was recently analyzed in rectal biopsies of treatment-naive pediatric patients with UC compared with healthy controls [71]. The data retrieved from patients with active UC revealed high expression levels of the clock genes *BMAL1*, *CLOCK*, *PER1*, *CRY1* and *RORα* and of the pro-and anti-inflammatory genes *IκB*, *IL-10*, *NF-κB1*, *NF-κB2*, *IL-6* and *TNF-α* [71]. In contrast, patients with active UC had low expression of the genes encoding mutual regulators of the circadian clock, metabolism, and inflammation, i.e., *RORγ*, *PGC1α*, *PPARα,* and *PPARγ* [71]. As the aforementioned metabolic and inflammatory regulators control clock gene expression and their expression is regulated by the circadian clock mechanism [91], it leads to a vicious cycle of complete circadian misalignment. Key results are summarized in Supplementary Table S1.

## Concluding remarks and future perspectives in IBD diagnosis and treatment

In this review, we bring evidence that the circadian clock, metabolism, and inflammation are intertwined, i.e., key factors in each system interact with the others, creating an intricate network. Despite some advancements in understanding the role of the internal clock in health and disease, a substantial gap remains between this molecular knowledge and its application in clinical practice. As previously discussed, considerable efforts have been made in recent years to bridge the results observed in experimental models to human contexts. However, the translation of this knowledge into clinical practice remains limited.

Clinical circadian research is needed to elucidate the mechanisms underlying the dysregulation of the AMPK-SIRT1-PGC1α metabolic axis in IBD and to evaluate the efficacy and safety of targeting this pathway as a therapeutic strategy. Similarly, strategies aimed at modulating circadian NF-κB activity with greater specificity and efficacy, while minimizing off-target effects, may lead to the development of safer and effective therapies for IBD. Combination therapies targeting multiple components of the inflammatory response together with medications targeting metabolic pathways, such as metformin, may offer greater efficacy and therapeutic benefit for patients with IBD. Moreover, personalized medicine approaches that take into account the individual chronotype, circadian variations in metabolic pathways, and immune functions may help tailor treatment strategies to specific patient subgroups and improve clinical outcomes. These experiments should increase our understanding of the importance of the circadian regulation of inflammation and metabolism and implement it in medical practice. Development of new tools to assess circadian system performance will assist us in implicating circadian parameters in clinical practice and in designing new therapies based on the regulation of the circadian system in order to increase the effectiveness of treatments. Salivary, sweat, or blood samples, which may be collected at several time points throughout the day, as well as data collected *via* wearable devices, may be useful to determine sleep-wake patterns, cardiovascular data, daily pattern of hormone secretion, and gene expression that connect the circadian system, metabolism, and inflammatory processes.

## Supplementary material

Online supplementary table S1

## Abbreviations

CD: Crohn’s disease
IBDs: inflammatory bowel diseases
PPARs: proliferator-activated receptors
RORs: retinoic acid receptor-related orphan receptors
SIRT1: Sirtuin 1
UC: ulcerative colitis
