# Supplementary material for: The circadian clock, metabolism, and inflammation—the holy trinity of inflammatory bowel diseases
Source: Clin Sci (Lond). 2025 Jul 4;139(13):777–90. doi: 10.1042/CS20256383 (PMC12312392; doi:10.1042/CS20256383)
Supplement: Online supplementary table S1 [file cs-139-13-CS20256383-s001.docx]

**Table S1: Summary of results linking clock, metabolism and inflammation.**

| **Study** | **Species/Model** | **Intervention** | **Outcomes** |
| --- | --- | --- | --- |
| AMPK phosphorylation of clock proteins | Cell culture | AMPK activation | CKIε activation; PER protein phosphorylation and degradation; CRY1 destabilization |
| AMPK effect on CRY1 stability | Cell culture | AMPK activation or overexpression | Phosphorylation of CRY1 by AMPK |
| Metformin and circadian rhythms | Mice | Metformin treatment (activates AMPK) | Altered circadian rhythm patterns |
| SIRT1 deacetylation of clock components | Cell culture | SIRT1 activation | Deacetylation of BMAL1 and PER2; enhanced PER2 degradation |
| PPARα agonism and PPARγ deletion | Mice | PPARα agonist treatment; PPARγ gene knockout | Altered circadian rhythms |
| REV-ERBα & RORα in circadian metabolism | Mice | Genetic manipulation of REV-ERBα and RORα | Changes in circadian behavior and metabolic gene expression |
| REV-ERBα knockout and adiposity | Mouse knockout (*Rev-erbα-/-*) | Rev-erbα gene deletion | Increased adiposity on normal and high-fat diet; enhanced fat uptake by adipose tissue |
| PGC1α circadian oscillation | Mice | Measure PGC1 levels over circadian cycle | PGC1 family (PGC1α) mRNA oscillates diurnally |
| PGC1α regulation of clock genes | Cell culture / mice | PGC1α overexpression or activation | Increased *Bmal1*, *Clock*, *Per2*, *Rev-erbα* expression |
| *Clock* mutant mice and metabolism | *Clock*Δ19 mutant mice | Loss-of-function mutation in Clock gene | Altered feeding pattern; obesity; metabolic syndrome |
| *Per2* knockout and diet-induced obesity | *Per2-/-* knockout mice | High-fat diet feeding | Marked obesity under high-fat diet |
| *Bmal1* knockout and metabolic rhythms | *Bmal1-/-* knockout mice | Genetic deletion of *Bmal1* | Loss of normal day-night triglyceride and glucose fluctuations; impaired gluconeogenesis; flattened daily insulin activity rhythm |
| Liver-specific *Bmal1* deletion | Conditional *Bmal1* knockout in hepatocytes | *Bmal1* deletion targeted to liver | Increased oxidative stress; elevated plasma triglycerides and cholesterol |
| Adipocyte-specific *Bmal1* knockout | Adipocyte-specific *Bmal1* knockout mice | *Bmal1* deletion in fat cells | Development of obesity |
| *Cry1/2-/-* effects | *Cry1-/-*;*Cry2-/-* mice | Genetic deletion of both *Cry1* and *Cry2* clock genes | Altered gluconeogenesis; increased susceptibility to obesity; hyperinsulinemia; enhanced adipose lipid storage; elevated pro-inflammatory cytokines |
| Circadian misalignment in humans | Human volunteers (controlled lab setting) | Forced circadian misalignment (desynchronized sleep-wake and meal cycles) | Decreased leptin; elevated blood glucose despite high insulin; blunted daily cortisol rhythm; increased blood pressure |
| Shift work and metabolic dysfunction | Human shift workers | Chronic night shift work (circadian disruption) | Impaired pancreatic β-cell function; reduced insulin sensitivity; impaired glucose tolerance; increased risk of type 2 diabetes and cardiovascular disease |
| Social jetlag and obesity risk | Human population cohort | Social jetlag | Higher body mass index (BMI); increased fat mass |
| Circadian control of immune cell trafficking | Mice | Observation of leukocyte migration across day-night cycle | Rhythmic oscillation in adhesion molecule expression (ICAM1, VCAM1, selectins); time-of-day dependent leukocyte tissue infiltration |
| Circadian regulation of macrophage cytokines | Mouse peritoneal macrophages | Isolations at different times of the day | Higher pro-inflammatory cytokine and chemokine expression at end of inactive phase |
| Time-of-day variation in infection susceptibility | Mice | Pathogenic infection or LPS challenge at different circadian times | Variable infection severity correlating with time of challenge; daily rhythm in NF-κB activation |
| NF-κB-clock interaction | Cell culture and genetically modified mice | Genetic or pharmacological disruption of NF-κB subunit p65 | Altered circadian gene expression period and amplitude when p65 is activated or inhibited |
| CLOCK protein and NF-κB signaling | Mouse model (CLOCK mutant) and cell studies | CLOCK deficiency; LPS-induced inflammation | Reduced NF-κB activation and lower pro-inflammatory cytokine induction in *Clock*-deficient mice after immune challenge |
| *Bmal1* knockout and immune cell rhythms | *Bmal1-/-* knockout mice | Genetic deletion of *Bmal1* | Abolished daily oscillations in circulating leukocytes and immature hematopoietic cells |
| Myeloid cell-specific *Bmal1* deletion | Myeloid lineage *Bmal1* knockout mice | Targeted removal of *Bmal1* in myeloid cells | Loss of daily fluctuations in inflammatory Ly6C^high^ monocytes in circulation |
| Neutrophil-specific *Bmal1* deletion | Neutrophil-specific *Bmal1* knockout mice | Targeted removal of *Bmal1* in neutrophils | No daily rhythm in neutrophil granule contents; impaired formation of neutrophil extracellular traps |
| REV-ERBα deficiency and macrophage inflammation | *Rev-erbα-/-* mice | *Rev-erbα* knockout | Hyper-responsive pro-inflammatory cytokine production in macrophages; loss of circadian IL-6 rhythm in response to LPS |
| RORα deficiency and inflammation susceptibility | RORα mutant mice (staggerer, RORα^sg/sg) | Genetic loss-of-function mutation in RORα | Heightened sensitivity to inflammatory challenge (LPS-induced lung inflammation); elevated neutrophil infiltration; increased IL-1β and IL-6 in bronchoalveolar fluid |
| RORα as a negative inflammation regulator | Cell culture / mice | Activation or overexpression of RORα | Increased IκBα expression; reduced NF-κB p65 nuclear translocation |
| Chronic shift work and systemic inflammation | Human night-shift workers vs day workers | Long-term rotating night shift | Elevated inflammatory markers (CRP, TNF-α, IL-6, IL-1β); increased WBC counts |
| Ramadan fasting and inflammation | Humans (healthy adults during Ramadan) | Intermittent fasting (dawn-to-sunset fasting during Ramadan) | Reduced pro-inflammatory cytokine expression (TNF-α, IL-6, IL-1β); lower circulating leukocyte counts |
| Time-restricted eating and inflammatory markers | Humans (adult participants, multiple studies) | Time-restricted eating (various schedules) vs habitual diet | Overall decrease in pro-inflammatory cytokine TNFα and leptin |
| Night shift work and IBD risk | Human epidemiological study | Night shift employment | Higher incidence of IBD among night-shift workers |
| Sleep disturbance and IBD activity | IBD patients | Self-reported sleep disruption and insomnia symptoms | More frequent disease flares and increased disease severity in patients with poor sleep |
| Clock gene expression in IBD patients | Human IBD patients (colonic biopsies and blood) | Measurement of clock gene mRNA levels in IBD vs controls | Reduced expression of BMAL1, CLOCK, PER1/2, CRY1/2 in inflamed intestinal tissue and peripheral WBCs of IBD patients |
| Circadian clock disruption and colitis severity | Mouse colitis models (clock gene mutants) | Genetic deficiency of clock components (Per1/2 double knockout, Rev-erbα mutant, global or intestinal Bmal1 knockout, RORα mutant) + DSS-induced colitis | Greater susceptibility to colitis: worsened inflammation, reduced goblet cells, impaired epithelial regeneration |
| Intestinal epithelial RORα knockout | Mice with intestinal epithelial cell-specific RORα deletion | Genetic knockout of RORα in gut epithelium | More severe colitis in DSS model; heightened NF-κB-driven inflammation (loss of RORα’s anti-inflammatory effect in gut lining) |
| BMAL1 and intestinal mucosal healing | BMAL1-deficient mice in colitis model | Genetic knockout of *Bmal1* (whole-body or intestinal) + DSS-induced colitis | Delayed recovery and wound healing of colonic epithelium; altered Wnt and Hippo signaling activity during regeneration |
| Clock genes and intestinal barrier function | *Clock* mutant and *Per2* knockout mice | Genetic disruption of *Clock* or *Per2*; assessment of colonic tight junction proteins | Tight junction proteins (occludin, claudin-1) oscillate inversely with *Per2* levels; PER2 knockout: consistently high occludin/claudin-1; CLOCK mutant: consistently low occludin/claudin-1 and increased intestinal permeability; greater susceptibility to DSS colitis in CLOCK mutant |
| PPARγ in ulcerative colitis | Human colon biopsy study | Analysis of PPARγ expression in intestinal tissue of UC patients vs controls | Significantly lower PPARγ expression in colonic epithelial cells of active UC patients |
| PGC1α overexpression and inflammation | Transgenic mice (liver-specific PGC1α overexpression) | PGC1α overexpression in hepatocytes | Enhanced anti-inflammatory IL-10 signaling effects; protection against diet-induced steatosis and insulin resistance |
| AMPK/SIRT1/PGC-1α pathway activation in colitis | Rats (acetic acid colitis) and human colon cell line | Pharmacological activation of AMPK-SIRT1-PGC1α axis | Down-regulation of pro-inflammatory cytokines; amelioration of colitis severity in rats |
